# The Influence of Life History and Sexual Dimorphism on Entheseal Changes in Modern Humans and African Great Apes

**DOI:** 10.1371/journal.pone.0107963

**Published:** 2014-09-24

**Authors:** Marco Milella

**Affiliations:** Anthropological Institute and Museum, University of Zurich, Zurich, Switzerland; Museo Nazionale Preistorico Etnografico ‘L. Pigorini’, Italy

## Abstract

Entheseal changes have been widely studied with regard to their correlation to biomechanical stress and their usefulness for biocultural reconstructions. However, anthropological and medical studies have demonstrated the marked influence of both age and sex on the development of these features. Studies of entheseal changes are mostly aimed in testing functional hypotheses and are mostly focused on modern humans, with few data available for non-human primates. The lack of comparative studies on the effect of age and sex on entheseal changes represent a gap in our understanding of the evolutionary basis of both development and degeneration of the human musculoskeletal system. The aim of the present work is to compare age trajectories and patterns of sexual dimorphism in entheseal changes between modern humans and African great apes. To this end we analyzed 23 postcranial entheses in a human contemporary identified skeletal collection (N = 484) and compared the results with those obtained from the analysis of *Pan* (N = 50) and *Gorilla* (N = 47) skeletal specimens. Results highlight taxon-specific age trajectories possibly linked to differences in life history schedules and phyletic relationships. Robusticity trajectories separate *Pan* and modern humans from *Gorilla*, whereas enthesopathic patterns are unique in modern humans and possibly linked to their extended potential lifespan. Comparisons between sexes evidence a decreasing dimorphism in robusticity from *Gorilla*, to modern humans to *Pan*, which is likely linked to the role played by size, lifespan and physical activity on robusticity development. The present study confirms previous hypotheses on the possible relevance of EC in the study of life history, pointing moreover to their usefulness in evolutionary studies.

## Introduction

Due to their supposed link with physical activity, morphological changes at the level of the entheses (skeletal attachment sites of muscles, ligaments and joint capsules), or entheseal changes (EC) [1] have been widely adopted as proxies of *in vivo* biomechanical stress in bioarchaeological studies [2], [3]–[8]. EC have indeed been used to test biocultural hypotheses about differences between contrasting socio-economic systems, possible presence of sexual subdivision of labour in past societies [9]–[14], and performance of specific daily physical activities [8],[15],[16]. Nonetheless, recent analyses of identified skeletal collections highlighted the multifactorial etiology of EC [17]–[23]. Sexual dimorphism [19],[22], anatomy of the attachment sites [21],[22], and, especially, age [17]–[24] appear strongly correlated with the expression of EC. Differences in the expression of EC in males and females has been observed in both archaeological and contemporary skeletal samples [14],[16],[19],[22],[23],[25]–[29], a pattern attributed to different activities performed by sexes and/or to the effect of physiological factors (e.g. hormones) on the expression of EC. About the relationship between the type of tissue of the attachment sites and EC, medical and anthropological studies pointed to substantial differences in terms of morphological variability between fibrous and fibrocartilaginous entheses (characterized, respectively, by the presence of dense fibrous connective tissue and fibrocartilage at the interface between bone and tendon [30],[31]) [21],[30],[32]. Such distinction has been recently adopted in anthropology, and considerations on the anatomy of entheses have been fully integrated in both the stages of data collection and discussion of results [20],[22],[32],[33]. Concerning age, both medical and anthropological studies agree on the strong role played by this variable on the expression of EC [17],[19]–[21],[23],[24],[34],[35], a complex phenomenon likely related to several causes, including the response of bone to continuous microtraumatic stress related to daily biomechanical stimuli, ontogenetic processes of the musculoskeletal system, and degenerative processes associated with aging.

Such results, besides suggesting a revision of previous functional hypotheses, allow placing EC in a wider perspective, establishing a link between these features and biological processes such as the evolution and development of the musculoskeletal system as well as shared and derived patterns in the life history of primates [19],[36].

Geometric properties of long bones [37]–[44], trabecular architecture of various skeletal regions [45]–[49], organization of postcranial muscles [50]–[52] and patterns of joint diseases, trauma and various pathological conditions [53]–[56] represent some of the main topics of anthropological research on the musculoskeletal system of primates and are mainly framed in a functional perspective. On the other hand, despite the wide array of studies on EC in modern humans, few data are available on EC in non-human primates, with only one publication [57] focusing on interspecific patterns of bilateral asymmetry and forelimb-hindlimb ratios, also in this case investigated in order to test their reliability in reflecting intertaxa behavioral patterns.

As far as life history is concerned, observations on the skeleton have been normally focused on early ontogenetic stages through the analysis of dental data [58]–[60] as well as cranial [61]–[64] and postcranial [64]–[68] developmental schedules. Topics of such studies include analyses of interspecific comparisons of skeletal growth patterns [64], differences in skeletal growth between captive and wild specimens [67], timing of first permanent molar emergence between wild great apes [58] and patterns of evolutionary developmental reorganization of the hominin skull [61]. Literature on skeletal changes associated to life history variables in the adult is, on the other hand, quite scanty, being represented by few studies mostly focused on aging patterns in single taxa or isolated skeletal samples [69]–[75]. Life history variables in the adult such as gestation length, interbirth interval, post-reproductive survivorship and differential longevity have been mostly studied on living specimens [76],[77], and rarely used in the discussion of skeletal data. However, clinical research has demonstrated the mutual physiological continuity between the muscular and skeletal systems and their sensibility to both endocrinal and metabolic factors [78]. Accordingly, it is expected that entheses, representing the interface between those systems, can be a good candidate for the study of skeletal changes related to life history variables in the adult. This was preliminary confirmed by the recent analysis of a large identified skeletal collection, which highlighted complex age- and sex-specific patterns in EC [19]. Starting from these premises, the aim of the present work is to contribute to the actual knowledge on EC across hominid taxa, by explicitly avoiding a functional perspective, and to explore their usefulness in the study of life history of primates and, more generally, in the analyses of evolutionary processes underlying both growth and aging of the musculosketal system. To this aim, we analyzed patterns of EC in a skeletal sample of modern humans and African great apes, testing the following hypotheses:

1. Due to interspecific differences in developmental schedules, interbirth interval and mean potential lifespan [61],[77],[79]–[82], we expect modern humans to exhibit diverging age trajectories of EC when compared with African great apes. Specifically, we expect a delayed development of entheseal structures in modern humans due to their delayed somatic growth when compared with African great apes [81],[83],[84].
2. Due to different levels of sexual dimorphism with regard to body mass and body size [85]–[87], we expect higher differences between sexes in entheseal development in *Gorilla* when compared with both modern humans and *Pan*.

## Materials and Methods

Our sample includes 484 modern humans, 50 *Pan*, and 47 *Gorilla* (Table 1). All specimens were selected on the basis of the absence of pathologies possibly affecting EC (Diffuse Idiopathic Skeletal Hyperostosis - DISH, and spondyloarthropathies) or the normal biomechanics of the body (fractures, dislocations, and dysplasias). DISH and spondyloarthropathies were diagnosed according Rogers & Waldron [88] and Martin-Dupont et al. [89]. Fractures, dislocations and dysplasias were diagnosed according to Lovell [90] and Ortner & Putschar [91]. The modern humans sample was selected from the identified skeletal collection Frassetto of Sassari [92], housed at the Museum of anthropology of the University of Bologna (Italy). It includes 274 male and 210 female subjects with documented age at death, sex, and profession from the beginning of the 20th century. Only subjects with age at death equal to or greater than twenty years old [19] were used in this study. Data on African great apes were collected at the Anthropological Institute of the University of Zurich (Switzerland) and the Royal Central African Museum of Tervuren (Belgium). The curators of the collections allowed access to the specimens and use of the relative datasets. Numbers of specimens, together with details on their age and sex can be found in Appendices S1–S2. The Zurich dataset includes 26 *Gorilla* (N males = 14, N females = 12) and 24 *Pan* (N males = 7, N females = 17), while the Tervuren dataset includes 21 *Gorilla* (N males = 9, N females = 12) and 26 *Pan* (N males = 15, N females = 11). The resulting sample includes both juvenile (erupted M2) and adult (erupted M3) individuals of both sexes (Table 1). While the *Gorilla* dataset includes mostly wild-shot specimens (only two captive individuals), 16 out of 50 *Pan* skeletal specimens are of captive individuals, mostly distributed in the first four age classes (Appendix S2). The choice to include in our dataset also captive animals was primarily dictated by the need to maximize the African great apes sample size. However, considering the different life history characterizing wild vs. captive individuals [65],[93], we decided to control for such possible source of bias in our analyses (see Methods section).

**Table 1:** Sample size, sex and age distribution of *Gorilla*, *Pan*, and *Homo*.

	*Gorilla*		*Pan*		*Homo*	
Age class	F	M	F	M	F	M
0	8	2	9	1	0	0
1	1	0	2	1	61	64
2	4	0	7	4	42	39
3	8	8	3	9	37	56
4	0	5	1	0	24	50
5	0	6	5	5	11	32
6	3	1	1	2	22	22
7	0	1	0	0	13	11
Total	24	23	28	22	210	274

F = females; M = males.

The modern humans sample was separated into classes of ten years each, for a total of seven age classes, while for African great apes we used seven stages of tooth wear (employing the first seven stages of Molnar [94] as a proxy for adult age, adding an eighth class – class “0”- for the juveniles, in order to distinguish them from the rest of the sample). We analyzed 23 postcranial entheses (Table 2) with regard to their development in robusticity (surface roughness) as well as proliferative (enthesophytes - EF) and resorptive (osteolytic - OL) entheseal changes (enthesopathies), according to the scoring schemes proposed by Mariotti et al. [17],[18]. Note that these methods, unlike others [32],[95] do not take into account the type of enthesis (i.e. fibrous vs. fibrocartilaginous), a factor often considered in studies on EC [2],[20]–[22],[96]. Nonetheless, it was chosen for: (a) the previous experience of the author with this method [19],[36],[97],[98]; (b) the site-specificity of the method for scoring robusticity (therefore indirectly taking into account the anatomy of each enthesis), and (c) the chance to consider separately different variables (robusticity and enthesopathies). We considered only the entheseal sites originally included in the method of Mariotti and colleagues [17]. This choice was driven (1) by the scoring criteria for robusticity, which are specific for each site, (2) by the need to analyze different sites in order to check for the expression of EC in the overall postcranial skeleton (3) by the need to compare data collected in different time frames.

**Table 2:** Entheses considered in the present study: anatomical location and relative functional complex.

Enthesis	Site	Functional complex
Costoclavicular lig.	clavicle	Shoulder
Conoid lig.	clavicle	
Trapezoid lig.	clavicle	
*M. pectoralis major*	clavicle (o)	
*M. deltoideus*	clavicle (o)	
*M. pectoralis major*	humerus (i)	
*M. latissimus dorsi/teres major*	humerus (i)	
*M. deltoideus*	humerus (i)	
*M. triceps brachii*	scapula (o)	Elbow (flexion/extension)
*M. brachioradialis*	humerus (o)	
*M. biceps brachii*	radius (i)	
*M. triceps brachii*	ulna (i)	
*M. brachialis*	ulna (i)	
*M pronator teres*	radius (i)	Forearm (pronation/supination)
Interosseous membrane	radius	
*M. supinator*	ulna (o)	
Enthesis	Site	Functional complex
*M. gluteus maximus*	femur (i)	Hip
*M. iliopsoas*	femur (i)	
*M. vastus medialis*	femur (o)	Knee
Quadriceps tendon	tibia	
Quadriceps tendon	patella	
*M. soleus*	tibia (o)	Foot
Achilles tendon	calcaneus	

M. = muscle; lig. = ligament; o = origin; i = insertion.

EF and OL were scored according to a four-degree scale [17]–[19]. In EF, this corresponds to a range from absence of proliferative patterns to the presence of obvious bony spurs. In OL, the scale ranges from absence of resorptive patterns to marked erosive areas at the level of entheses. In analyzing our data, we followed the protocol detailed in Milella et al. [19] although applying some minor changes. In regression analyses (see below) we used the original four-degree scale for each feature. On the other hand, in pairwise comparison we considered for EF only the presence of obvious proliferative patterns (degrees 2 and 3) while, for OL, the complexity of these features suggested to consider their absence (0), the presence of fine porosities (“pitting” - degree 1) and the presence of extensive osteolytic areas (“erosions” - degrees 2 and 3) [19]. Robusticity was scored according to a five-degree scale (from 0 – extremely weak development– to 4 – marked development). In analysing robusticity, we assumed that each degree represents an underlying continuous variable. Accordingly, in order to explore general patterns of robusticity we grouped the attachment sites into eight functional complexes (Table 2) and calculated a mean robusticity score (MRS) for each complex (see for details Mariotti et al. [17] and Milella et al. [19], and, for a similar approach, Niinimäki [96]). Qualitative scores like the one used in this study are typically affected by varying degrees of intra- and interobserver error. The method of Mariotti and colleagues for the scoring of EF and OL is reported to be associated with a good (less than 5 percent) intra- and interobserver error for both EF and OL [18]. About robusticity, Mariotti and colleagues report intraobserver and interobserver error percentages of, respectively, 20 and 28 after lumping the first three degrees of robusticity. In this study, we decided to check the intraobserver error for both enthesopathies (EF and OL) and mean robusticity scores (MRS). For the interobserver error, we will discuss the values published by Mariotti and colleagues [17],[18] on the basis of our results.

Since a study of bilateral asymmetry is beyond the scope of this work, all analyses were performed only for the left side (chosen in order to maximize the sample size).

Our analyses included:

1. A correlation test between age and EC (EF, OL and MRS) through the Spearman rank sum test.
2. A test of sexual differences in EC through intra-group pairwise comparison of MRS (Wilcoxon test) and frequencies of EF and OL (chi squared test).

Given the small size of the African great apes sample, step 1) was computed by pooling together the sexes, while in step 2) we grouped together the age classes 1–7, excluding the juvenile specimens.

In order to calculate the intraobserver error in the scoring of EF, OL, and MRS, we collected EC data on 58 specimens (20 modern humans, 20 *Pan*, and 18 *Gorilla*) twice, in two independent sessions. The percentage of disagreement between the two sessions and the relative Cohen’s weighted kappa was used for calculating the intraobserver error in the scoring of EF and OL. Intraobserver error in the scoring of MRS was calculated for each complex by comparing the scores from the two sessions with the Wilcoxon test.

Finally, due to the possible bias introduced on the correlation between EC and age by the presence in the *Pan* sample of a relatively large proportion of captive individuals, we decided to compare the results obtained by analyzing separately captive and wild-shot individuals. A similar comparison was not considered necessary for the *Gorilla* sample, given the small percentage of captive individuals in this group (2/47: 4.3%).

All statistical analyses were performed with JMP 10.0.0 (SAS Institute Inc. 2012) setting alpha to 95%. Cohen’s weighted kappa was calculated with the package psych (version 1.4.5) [99] in R 3.0.3 [100].

## Results

### Intraobserver error

The intraobserver error for EF and OL is acceptable, with error frequencies of, respectively, seven and six percent. The Wilcoxon test performed on the MRS data of the two scoring session reflect overall a negligible disagreement for all the functional complexes (Table S1).

### Age

Tables S2–S4 show the summary statistics for EF, OL, and MRS in our sample. Original data for each variable can be found in Appendices S3–S5.

In testing interspecific differences in EC age trajectories, we first analyzed only adult individuals, and in a second phase, we included African great apes juvenile specimens.

In *Pan*, correlation tests between EC and age in captive and wild-shot *Pan* evidence a general lack of differences between these two groups (Tables S5–S7), justifying their inclusion in a single sample.

**Figure 1:**
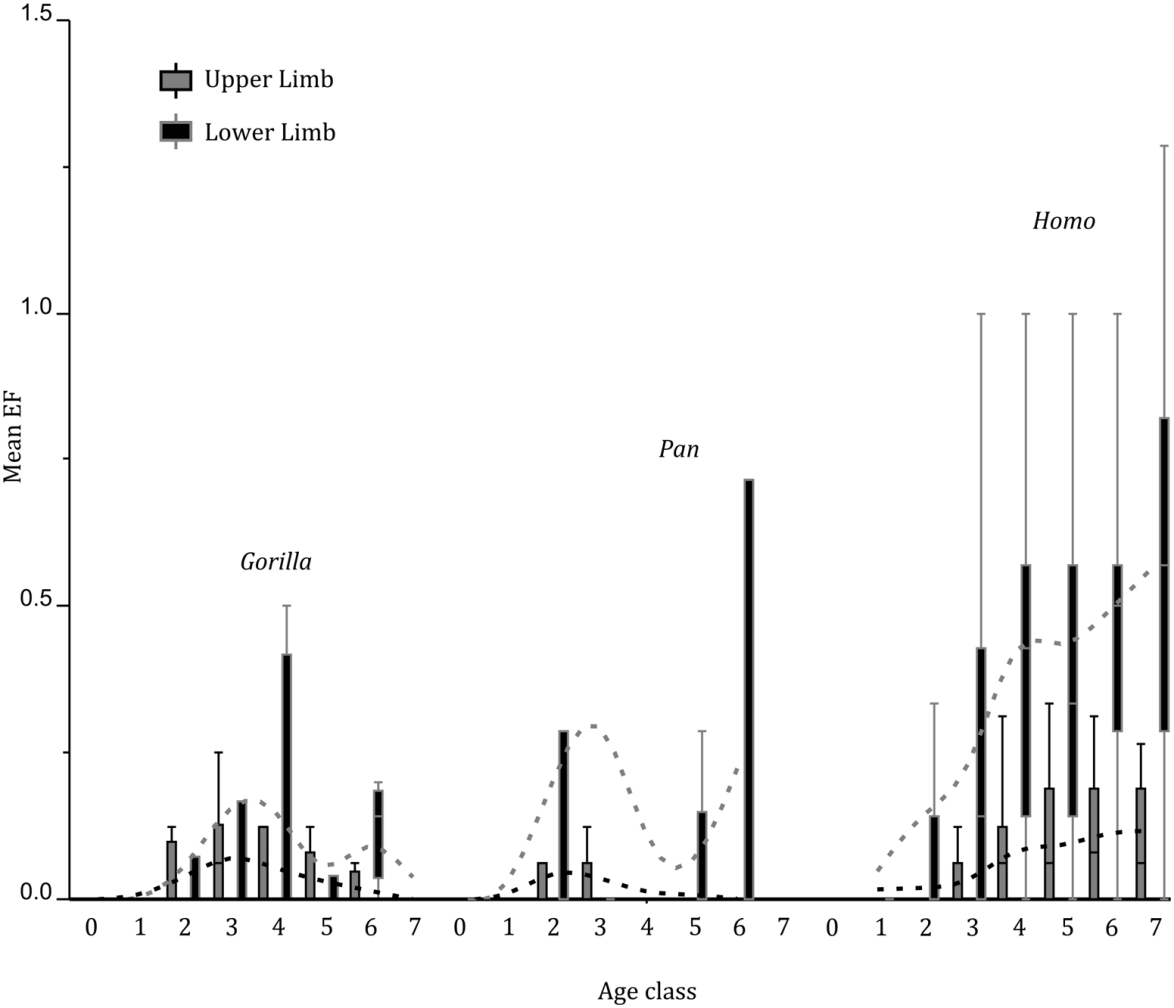
Box plots of mean EF vs. age. Note the clear increase with age of EF in modern humans, and the lack of clear patterns in African great apes.

When considering only adult specimens, modern humans are characterized by a positive correlation between EF and age, which is significant for a large number of sites. On the other hand, no significant correlation was found in African great apes between EF and age (Table S8; see also Figure 1). Regarding OL, both positive and negative correlations with age are seen in modern humans (Table S9), a pattern that is consistent with earlier analyses [19] showing a complex pattern of OL, with some sites characterized by different age trajectories of degree 1 and degrees 2 and 3. African great apes are characterized overall by a negative correlation between OL and age. OL correlation with age characterizes a limited suite of sites, only partially overlapping between taxa (Table S9). Interestingly, no differences in age trajectories between OL degrees are present in both *Pan* and *Gorilla*. The addition of juvenile specimens does not alter the age patterns in both types of enthesopathic changes (see also Figure 1).

As far as robusticity is concerned, results show a significant positive correlation between age and MRS in both *Gorilla* and *Homo*, whereas in *Pan* a similar trend is shown only by complexes of the upper limb and by the foot (Table S10). When comparing the hindlimb and forelimb correlation with age, African great apes show on average higher correlation values for the latter, whereas in modern humans no clear difference is evident between the upper and lower limbs.

**Figure 2:**
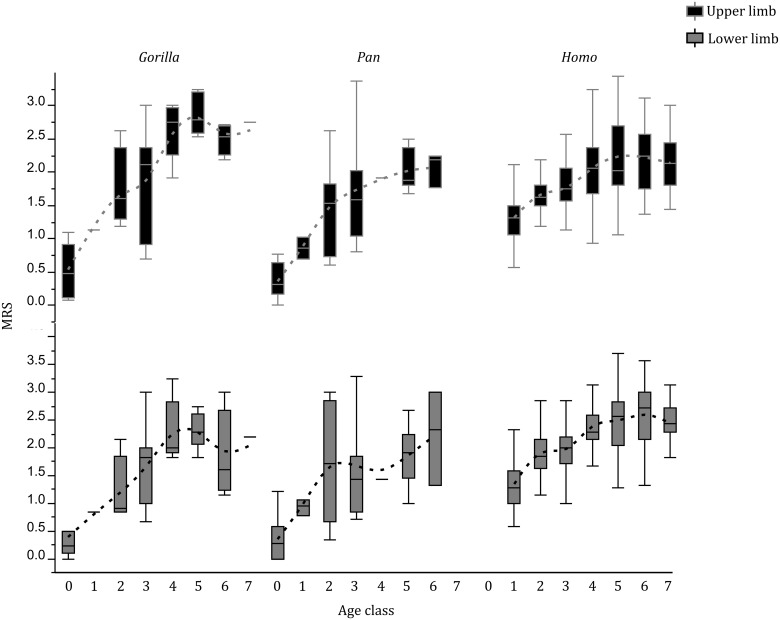
Box plots of mean robusticity score (MRS) vs. age. Note the steep and regular increase of MRS in *Gorilla*, and the more complex trajectories shared by *Pan* and modern humans.

**Figure 3:**
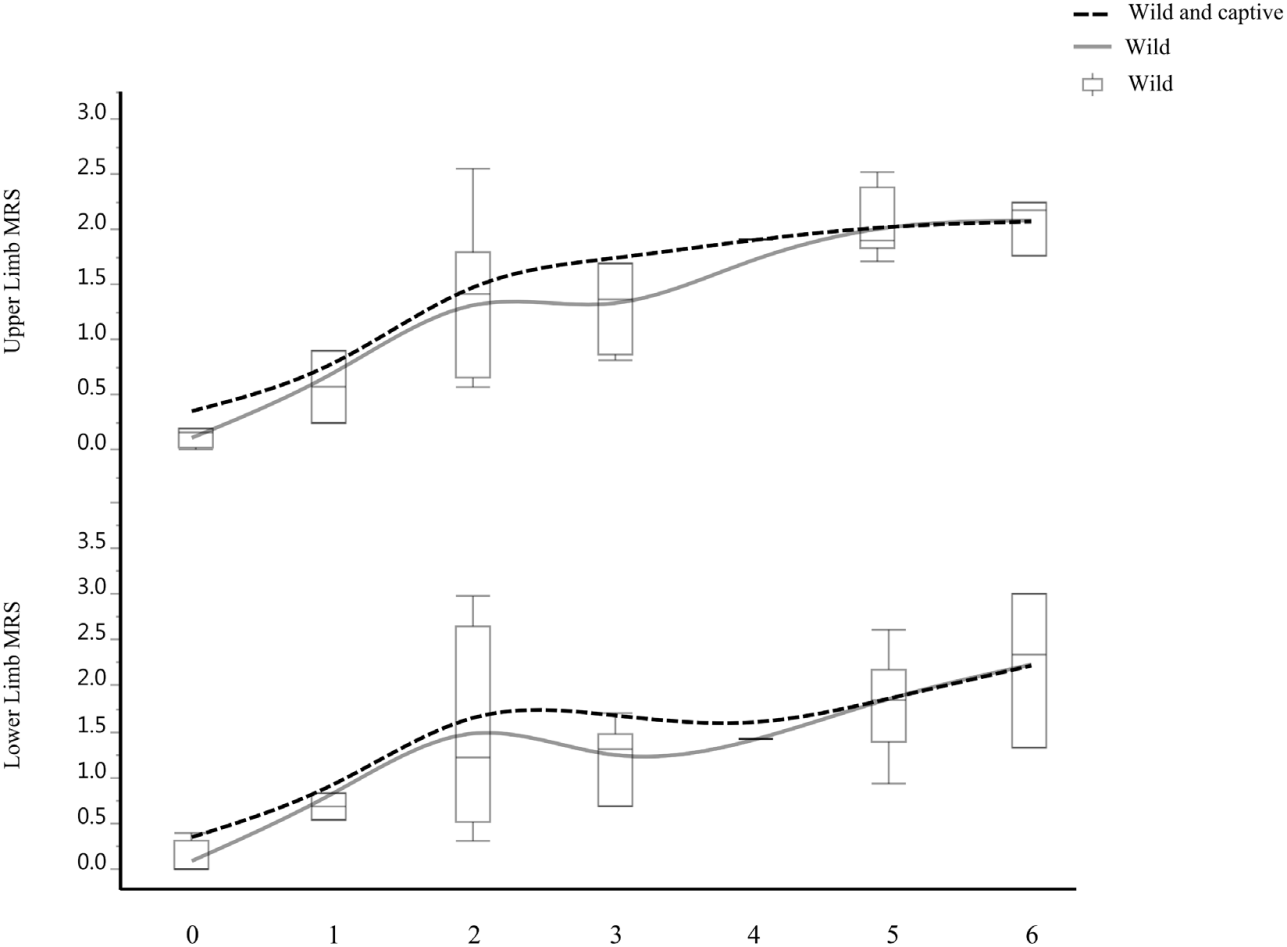
*Pan*: mean robusticity score (MRS) vs. age after excluding the captive specimens (grey line and box plots) compared with the age trajectories of the complete *Pan* sample (dotted black line, see also **Figure 2**). Note the increase in complexity of the age trajectories characterizing the wild specimens.

MRS age trajectories and rates of MRS increase by age class are specific among taxa (Figure 2). These include: (a) a fast increase until the fourth age stage in *Gorilla* followed by a sudden decrease; (b) a similar increase in *Pan* until the third age phase, starting from which, both the upper and lower limbs show a less pronounced increase of MRS with advancing age and (c) an increase of MRS in modern humans during the first two age phases (until forty years old) followed by a further growth that reaches a peak around the fifth age phase (between sixty and sixty nine years old) and, starting from the sixth age phase, a slight decrease. When adding the juvenile specimens to the African great apes sample results in a more marked correlation between age and MRS, a pattern that is particularly evident in *Pan* and, overall, represented by steeper age trajectories in both taxa. Comparing such trajectories with those characterizing modern humans, the latter show similar trends, but shifted by about a decade. When excluding from the *Pan* sample the captive specimens, results show a more complex age trajectory of MRS, with an increase of MRS along the first two age phases followed by a stasis and a further increase starting from the fourth age class (Figure 3).

### Sexual dimorphism

Given the observed correlation between age and EC we did a preliminary check for age differences among taxa (only adult specimens were considered in testing for EC sexual dimorphism). Intersex comparison for mean age in modern humans (by a Student t test) and sample size of each age class in *Pan* (by a Chi square test) resulted in no significant differences. In *Gorilla,* the age classes 2, 4, and 5 have different sample sizes between sexes, resulting in males being on average older than females, and, therefore, analyses of sexual dimorphism in this taxon are more prone to be biased by the effect of age. Despite this issue, we preferred to compare sexes without subdividing the sample into age classes for all taxa, and to consider the possible effect of age on *Gorilla* sexual dimorphism when discussing our results. In modern humans significant differences between the sexes in EF are limited to the lower limb, with males showing higher frequencies at the mm. *quadriceps femoris* (patellar site) and *soleus.* In the African great apes sample, a test of differences between the sexes for EF was possible only for the ulnar enthesis of the m. *tricipes brachii* (*Gorilla* and *Pan*) and for the quadriceps tendon on the patella (*Gorilla*), which overall show a lack of differences between sexes (Table S11). For OL, these are more present in males for the costoclavicular ligament, the m. *biceps brachii*, and for the m. *pectoralis major* (humeral site). The same pattern is present in *Gorilla* for the costoclavicular ligament, while no sexual differences were found in *Pan* (Table S12). Analysis of sexual dimorphism in MRS reveals higher development in modern human males for the elbow, forearm, hip, and for the overall upper limb. In *Gorilla,* males show higher development at the elbow, knee, and for the overall upper and lower limbs, while no sexual dimorphism was observed in *Pan* (Table S13).

## Discussion

### Intraobserver error

Intraobserver error for the scoring of EF, OL, and MRS reflect a good consistency among our data. Interobserver errors for EF and OL have been reported to correspond to less than 5% [18], whereas for robusticity the same error (but considering only three robusticity degrees) has shown to reach 20% [17]. In our case no significant intraobserver disagreement was found for MRS. At the same time a calculation of intraobserver error of robusticity scores for single sites gives a value of 41%, but with a root mean squared error of only 0.9. In fact, most disagreements are due to differences in scoring of just one degree of robusticity, as already noted by Mariotti and colleagues [17]. Such results stress the advantage of using functional mean robusticity scores (MRS) instead of raw data from single sites, and, at the same time, suggest that the interobserver reported in the literature [17] for this variable would be markedly lower when using MRS.

### Age

Our first hypothesis postulated taxon-specific age trajectories of EC, and, specifically, a delayed increase of EC with age in modern humans when compared with African great apes.

This hypothesis was partially confirmed by our results, which show: (a) a linear increase of MRS with age in *Gorilla,* (b) a more complex and, starting from the third age phase, more moderate increase in *Pan* and modern humans, and, (c) a delayed increase of MRS growth as a function of absolute age in modern humans. Such results are consistent with interspecific differences in growth rate [77],[81],[84],[101],[102]. In *Gorilla*, the high rate of MRS increase in this taxon and its delayed decrease when compared with *Pan* can be traced back to Gorillas’ fast somatic growth [77] and, at least in males, a later slowing of growth when compared with *Pan* [101]. Age trajectories of *Pan* and modern humans are more complex. The high rate of MRS increase in *Pan* during early age overlaps that observed in *Gorilla* and is consistent with the faster somatic growth of this taxon compared to modern humans [77]. In both *Pan* and modern humans the MRS trajectories are similar and characterized by an increase until the second age phase, a stasis until the third, and a further increase. Such results are further strengthened when considering only wild *Pan* specimens. The resulting trajectories are indeed more complex and similar to those of modern humans, with the presence of a marked stasis in MRS increase starting from the second age phase. Such pattern can be linked to the exclusion of captive specimens, which, due to their faster somatic growth [65],[93], are likely to bias the MRS patterns when added to the full dataset. Beside the possible biomechanical factors influencing the observed MRS trajectories, the closeness of the MRS age trajectories of *Pan* and modern humans, and their specificity when compared with those of *Gorilla* raise questions about the possible evolutionary meaning of this pattern. More specifically, it mirrors previous arguments about derived vs. shared life history traits in modern humans compared with African great apes in the literature [81]. Overall, our results are not consistent with a unique pattern of development and aging in modern humans. On the other hand, the possibility of parallel evolution of these traits in modern humans and *Pan* seems difficult to accept considering the different types of biomechanical stress (i.e. locomotory patterns) experienced by these taxa as well as the dissimilarity of other variables possibly affecting MRS development (e.g. body size, diet). Accordingly, it is plausible to postulate between modern humans and *Pan* a suite of synapomorphic patterns in the development of their musculoskeletal system in common with their last common ancestor.

Results on enthesopathies show: (a) an increase of EF with age only in modern humans, (b) different trajectories of OL in modern humans and African great apes, but a partial overlap between taxa of the sites showing correlation between OL and age. The lower frequencies of EF in African great apes can be compared with previous works [53],[103] which showed low frequencies of degenerative joint disease in great apes. Such results, besides being possibly correlated to interspecific differences in locomotory patterns (e.g. the marked development of EF at the level of the Achilles tendon in modern humans and not in African great apes), should be considered as a function of age. The positive correlation of EF with age in modern humans and their higher frequency in elderly subjects [19],[97] indeed points to interspecific differences in maximum potential lifespan [82] as the main factor underlying the observed patterns. This seems further confirmed by clinical research showing a degenerative origin of these EC [24],[104],[105]. Accordingly, the lack of correlation between EF and age in African great apes could be linked to a shorter lifespan accompanied by a similar (or even slower) rate of degenerative processes when compared with modern humans. Results on OL are consistent with the presence in African great apes of remodeling patterns at the level of entheses similar to what has already been observed during growth in modern humans [18],[19],[106]–[108]. On the other hand, the increase of porosities with age in modern humans and its lack in African great apes is, as for EF, consistent with degenerative processes (see [18],[19],[109]) specific to modern humans and mostly related to their longer lifespan.

Recent analyses of mountain gorillas [68] evidenced patterns of long bone strength consistent with ontogenetic changes in locomotory behavior. In our study, the small sample size led us to group African great apes only at the level of genus, a fact that hampers fine-grained analyses of specific ontogenetic patterns. However, the distinct age trajectories described by MRS, and the partial correlation showed between EC and long bones geometric properties [110] make the extension of our study to the interspecific level a promising line of future research.

### Sexual dimorphism

Our second hypothesis postulated higher entheseal changes in males *Gorilla* when compared with both modern humans and *Pan*. This was only partially confirmed by our results, and specifically only by sexual differences in MRS (Table S13), which evidence a scenario where *Gorilla* and modern humans appear markedly dimorphic when compared with *Pan.* Conversely, when comparing interspecific degrees of MRS sexual dimorphism, *Gorilla* shows a more pronounced difference between sexes. Even recognizing the possible influence on this result of the different age distribution between sexes in *Gorilla* (see above and Table 1), our data are interestingly consistent with previous studies comparing degrees of sexual dimorphism among great apes [86]. Specifically, the intermediate position of modern humans between *Gorilla* and *Pan* is consistent with the results of Lovejoy and colleagues [111],[112] on articular dimensions. The higher sexual dimorphism of modern humans when compared with *Pan* can be traced back to a marked dimorphism in body size in the human population used in this study. EC have been indeed found to correlate with size [25],[26],[113]. Note moreover that the type of sexual dimorphism observed in our dataset is consistent with previous studies on EC [6],[15],[22],[114]. As an alternative (or possible complementary) explanation, note that previous analyses on the same human dataset [19] evidenced a marked sexual dimorphism starting from fifty years old. It is therefore possible that, as for age trajectories, the different degree of sexual dimorphism observed in modern humans and *Pan* is partially correlated with the longer lifespan in modern humans. An additional factor could be a marked subdivision of physical activities between the sexes in the population of Sassari. This, coupled with a longer lifespan, could have led to a higher expression of differences between sexes.

Concerning EF and OL, note that intersex comparisons in *Gorilla* and *Pan* are strongly hampered by both the small sample sizes of these taxa and the general poor expression of EF in our African great apes sample (see above). It is therefore possible that different results could be obtained by analyzing a larger dataset of both taxa. It is moreover possible that the grouping in our intersex analyses of all age classes masks patterns of sexual dimorphism more nuanced than the ones observed in MRS. Both EF and OL, when controlling for age, show complex (though not obvious) differences between sexes in modern humans [19],[97].

## Conclusions

In the present study, we compared age trajectories and patterns of sexual dimorphism in entheseal robusticity and enthesopathic development between modern humans, *Pan* and *Gorilla*. Intraobserver error for the scoring of enthesopathies and MRS is overall low, pointing to a good reliability of our data.

Results revealed specific age trajectories among taxa that can be interpreted both in terms of different life history schedules and phyletic relationships. Robusticity trajectories separate *Pan* and modern humans from *Gorilla*, whereas modern humans show unique patterns of enthesopathic development which are likely the result of their extended potential lifespan. As far as sexual dimorphism is concerned, our results suggest the possible relevance of size, lifespan and physical activity in expressing differences between sexes, stressing on the other hand the need for larger samples in order to better check patterns of sexual dimorphism in enthesopathic development The present study confirmed previous hypotheses [19] on the possible relevance of EC in the study of life history, pointing moreover to their usefulness in evolutionary studies.

## Supporting Information

Table S1 a) Results of the Wilcoxon test for the intraobserver error in the scoring of mean robusticty scores between two independent sessions: b) Percentage of intraobserver disagreement and Cohen’s weighted kappa for EF and OL.
(XLS)

Table S2 **Proliferative enthesopathies (EF): summary statistics by genus and age class.** SD = standard deviation.
(XLS)

Table S3 **Osteolythic enthesopathies (OL): summary statistics by genus and age class.** SD = standard deviation.
(XLS)

Table S4 **Mean robusticity score (MRS) summary statistics by genus and age class.** SD = standard deviation.
(XLSX)

Table S5 ***Pan*** **: Comparison between captive (C) and wild-shot (W) specimens in correlation between proliferative enthesopathies (EF) and age.** Empty cells indicate cases for which the Spearman’s test was not possible.
(XLS)

Table S6 ***Pan*** **: Comparison between captive (C) and wild-shot (W) specimens in correlation between osteolithic formations (OL) and age.** Empty cells indicate cases for which the Spearman’s test was not possible.
(XLS)

Table S7 ***Pan*** **: comparison between captive (C) and wild-shot (W) specimens in correlation between mean robusticity scores (MRS) and age.**
(XLS)

Table S8 **Proliferative enthesopathes (EF): correlation with age.** Empty cases indicate sites for which the Spearman’s test was not possible. Analyses in *Gorilla* and *Pan* include both adults and juvenile specimens. ns = not significant.
(XLS)

Table S9 **Osteolythic enthesopathies (OL): correlation with age.** Empty cases indicate sites for which the Spearman’s test was not possible. Analyses in *Gorilla* and *Pan* include both adults and juvenile specimens. ns = not significant.
(XLS)

Table S10 **Correlation between mean robusticity scores (MRS) and age before and after the inclusion to the Gorilla and Pan samples of juvenile specimens.** ns = not significant.
(XLS)

Table S11 **Proliferative enthesopathies (EF): sex differences in the frequencies (%) of degree (2+3).** F = females; M = males. Empty cases indicate sites for which the chi suqred test was not possible. ns = not significant.
(XLS)

Table S12 **Osteolythic enthesopathies (OL): sex differences in the frequencies (%) of degrees (1) and (2+3).** Empty cases indicates sites for which the chi squared test was not possible. F = females; M = males. ns = not significant.
(XLS)

Table S13 **Sex differences (Wilcoxon test) of Mean Robusticity Scores (MRS).** F = females;M = males; ns = not significant.
(XLS)

Appendix S1 **List of human specimens used in this study.** MAUB: Museum of Anthropology, University of Bologna, Italy.
(XLS)

Appendix S2 **List of** ***Gorilla*** **and** ***Pan*** **specimens used in this study.** RMCA: Royal Museum of Central Africa, Tervuren, Belgium; AIM: Anthropological Institute and Museum, University of Zurich, Switzerland.
(XLS)

Appendix S3 **Proliferative enthesopathies (EF): complete dataset.** Empty cases indicate missing data.
(XLSX)

Appendix S4 **Osteolythic enthesopathies (OL): complete dataset.** Empty cases indicate missing data.
(XLSX)

Appendix S5 **Mean robusticity scores (MRS): complete dataset.** Empty cases indicate missing data.
(XLS)
